# Comprehensive Analysis of Germline Variants in Mexican Patients with Hereditary Breast and Ovarian Cancer Susceptibility

**DOI:** 10.3390/cancers10100361

**Published:** 2018-09-27

**Authors:** Rosalía Quezada Urban, Clara Estela Díaz Velásquez, Rina Gitler, María Patricia Rojo Castillo, Max Sirota Toporek, Andrea Figueroa Morales, Oscar Moreno García, Lizbeth García Esquivel, Gabriela Torres Mejía, Michael Dean, Iván Delgado Enciso, Héctor Ochoa Díaz López, Fernando Rodríguez León, Virginia Jan, Víctor Hugo Garzón Barrientos, Pablo Ruiz Flores, Perla Karina Espino Silva, Jorge Haro Santa Cruz, Héctor Martínez Gregorio, Ernesto Arturo Rojas Jiménez, Luis Enrique Romero Cruz, Claudia Fabiola Méndez Catalá, Rosa María Álvarez Gómez, Verónica Fragoso Ontiveros, Luis Alonso Herrera, Isabelle Romieu, Luis Ignacio Terrazas, Yolanda Irasema Chirino, Cecilia Frecha, Javier Oliver, Sandra Perdomo, Felipe Vaca Paniagua

**Affiliations:** 1 Laboratorio Nacional en Salud, Diagnóstico Molecular y Efecto Ambiental en Enfermedades Crónico-Degenerativas, Facultad de Estudios Superiores Iztacala, Tlalnepantla, Estado de México 54090, Mexico; rosieurban@hotmail.com (R.Q.U.); cdiazvelasquez@aol.com (C.E.D.V.); mag_hector@hotmail.com (H.M.G.); erarroji@gmail.com (E.A.R.J.); k.ikeo3@comunidad.unam.mx (L.E.R.C.); mendezcatalacf@gmail.com (C.F.M.C.); literrazas@unam.mx (L.I.T.); irasemachirino@gmail.com (Y.I.C.); 2 Fundación Alma, CDMX 11560, Mexico; direccion@alma.org.mx (R.G.); mpatrojoc@gmail.com (M.P.R.C.); maxsir89@gmail.com (M.S.T.); eidna.92@gmail.com (A.F.M.); ojmor10@gmail.com (O.M.G.); liz.gaes@gmail.com (L.G.E.); 3 Instituto Nacional de Salud Pública, 62100 Cuernavaca, Morelos, Mexico; gtorres@insp.mx; 4 Division of Cancer Epidemiology and Genetics, National Cancer Institute, Bethesda, MD 20892, USA; deanm@mail.nih.gov; 5 Instituto Estatal de Cancerología de Colima, Colima 28000, Colima, Mexico; ivancoliman@hotmail.com; 6 Department of Health, El Colegio de la Frontera Sur (ECOSUR), San Cristóbal de Las Casas 29290, Chiapas, Mexico; hochoa@ecosur.mx (H.O.D.L.); ferrodleon@hotmail.com (F.R.L.); 7 Internal Medicine, Hospital de Especialidades Vida Mejor, ISSTECH, Tuxtla Gutiérrez 29040, Chiapas, Mexico; viky_jan@hotmail.com; 8 Hospital General de Chilpancingo, Chilpancingo de los Bravo 39019, Guerrero, Mexico; victhorgb@gmail.com; 9 Centro de Investigación Biomédica, Universidad Autónoma de Coahuila, Torreón 27000, Coahuila, Mexico; pabloruiz1047@gmail.com (P.R.F.); pekaessi@hotmail.com (P.K.E.S.); qfbharosanta@gmail.com (J.H.S.C.); 10 Instituto Nacional de Cancerología, CDMX 14080, Mexico; rosamag2@hotmail.com (R.M.Á.G.); ontiverosfvero@yahoo.com.mx (V.F.O.); 11 Unidad de Investigación Biomédica en Cáncer, Instituto de Investigaciones Biomédicas-Instituto Nacional de Cancerología, CDMX 14080, Mexico; metil@hotmail.com; 12 Center for Center for Research on Population Health, National Institute of Public Health, Cuernavaca 62100, Morelos, Mexico; iromieu@gmail.com; 13 Hubert Department of Global Health, Emory University, Atlanta, GA 30322, USA; 14 Unidad de Biomedicina, Facultad de Estudios Superiores Iztacala, UNAM, 54090 Tlalnepantla, Estado de México, Mexico; 15 Hospital Italiano, Buenos Aires C1199ABB, Argentina; frechacecilia@gmail.com (C.F.); javiom@gmail.com (J.O.); 16 Investigación en Nutrición, Genética y Metabolismo, Facultad de Medicina, Universidad El Bosque, Bogotá 110121, Colombia; perdomosandra@unbosque.edu.co; 17 Department of Pathology and Laboratories, Hospital Universitario Fundación Santa Fe de Bogotá, Bogotá 110100, Colombia

**Keywords:** *BRCA1/2*, genetic screening, hereditary breast cancer, massive parallel sequencing, gene panel, pathogenic variants

## Abstract

Hereditary breast and ovarian cancer syndrome (HBOC) represents 5–10% of all patients with breast cancer and is associated with high-risk pathogenic alleles in *BRCA1/2* genes, but only for 25% of cases. We aimed to find new pathogenic alleles in a panel of 143 cancer-predisposing genes in 300 Mexican cancer patients with suspicion of HBOC and 27 high-risk patients with a severe family history of cancer, using massive parallel sequencing. We found pathogenic variants in 23 genes, including *BRCA1/2*. In the group of cancer patients 15% (46/300) had a pathogenic variant; 11% (33/300) harbored variants with unknown clinical significance (VUS) and 74% (221/300) were negative. The high-risk group had 22% (6/27) of patients with pathogenic variants, 4% (1/27) had VUS and 74% (20/27) were negative. The most recurrent mutations were the Mexican founder deletion of exons 9-12 and the variant p.G228fs in *BRCA1*, each found in 5 of 17 patients with alterations in this gene. Rare VUS with potential impact at the protein level were found in 21 genes. Our results show for the first time in the Mexican population a higher contribution of pathogenic alleles in other susceptibility cancer genes (54%) than in *BRCA1/2* (46%), highlighting the high locus heterogeneity of HBOC and the necessity of expanding genetic tests for this disease to include broader gene panels.

## 1. Introduction

Breast cancer (BC, OMIM#114480) is the most prevalent cancer in the world and accounts for 14.7 million of mortality cases [1]. Approximately, 10% of BC cases have a genetic, inherited etiology referred as Hereditary Breast and Ovarian Cancer (HBOC) with an important impact in genetic counseling and cancer prevention interventions [2].

Pathogenic variants in *BRCA1* and *BRCA2* genes are the most prevalent in HBOC, collectively contributing to 15–25% of the cases [3]. Pathogenic alleles in these genes frequently have high penetrance and have been found in different populations, including countries from Latin America, such as Mexico, Colombia, Argentina, Chile, Brazil, among others [4,5]. However, locus heterogeneity has been found in patients without mutations in *BRCA1* and *BRCA2* [6] together with additional pathogenic variants at lower frequency and in genes that confer moderate risk including *CHEK2*, *PALB2*, *ATM*, *FANCM*, *ATR*, *STK11*, *RAD51C*, *BRIP1*, *CDH1*, *NF1*, *NBN* and *ERCC3* [7,8]. The prevalence of these novel, moderate-risk genes in HBOC patients has recently started to be defined by massive parallel sequencing (MPS). Studies indicate that causal variants have very low frequency in most of the populations studied and are spread in a larger array of genes that remain unexplored (Table 1). Until now, the contribution of pathogenic variants in genes other than *BRCA1* and *BRCA2* has not been entirely defined and studies in Latin American populations are still scarce.

In recent years, important efforts to define common susceptibility loci for breast cancer in large cohorts have identified more than 90 SNPs, which predispose to this disease [26]. However, the risk conferred by these common susceptibility loci can explain up to 14% of hereditary breast cancer aggregation in the European population [27]. Additional SNPs remain to be discovered and association studies need to be conducted in other populations to better define the prevalence and clinical relevance of novel pathogenic alleles [28]. The identification of rare or population specific, high/moderate-risk pathogenic alleles could be translated into better molecular diagnosis, personalized risk assessment and treatment [23].

To determine the prevalence of pathogenic variants in cancer predisposing genes in Mexican patients, an understudied mixed population, and the potential benefit for molecular diagnosis with gene panel testing, we performed a germline genetic analysis in 327 patients with a clinical indication of HBOC. We analyzed all cases using a panel of 143 genes associated with different inherited oncologic diseases, by massive parallel sequencing.

## 2. Results

### 2.1. Clinical and Epidemiological Description of Breast Cancer Cases

Clinical and pathological characteristics of a total of 300 sequenced cases diagnosed with breast cancer are described in Table 2. Mean age at diagnosis was 41 years (range 23–69, SD: 7.3). Seventy one percent of cases had a family history of cancer, 85% reported at least one pregnancy and the average parity was 3 children (SD: 1.6), 60% never used oral contraceptives and 93% reported not being current alcohol drinkers. Importantly, sixty two percent of all cases were overweight, obese or extremely obese. Mutational status was defined as the presence of a pathogenic or likely pathogenic variant (American College of Medical Genetics and Genomics classification) in any of the 143 genes evaluated [29]. Fifteen percent of this group had a pathogenic or likely pathogenic variant. 

Age at diagnosis was the only epidemiological characteristic statistically associated with mutational status (*p* = 0.04). No association was found between stage, histological subtype, hormone receptor status and mutational status in cases. Analysis by individual gene showed no association between presence of a mutation and a clinical or pathological characteristic.

Patients in the older age group (60–69 years) were characterized by presenting with early stage tumors (I/II) and absence of mutations.

### 2.2. Pathogenic Variants in the Breast Cancer Cases

**Figure 1:**
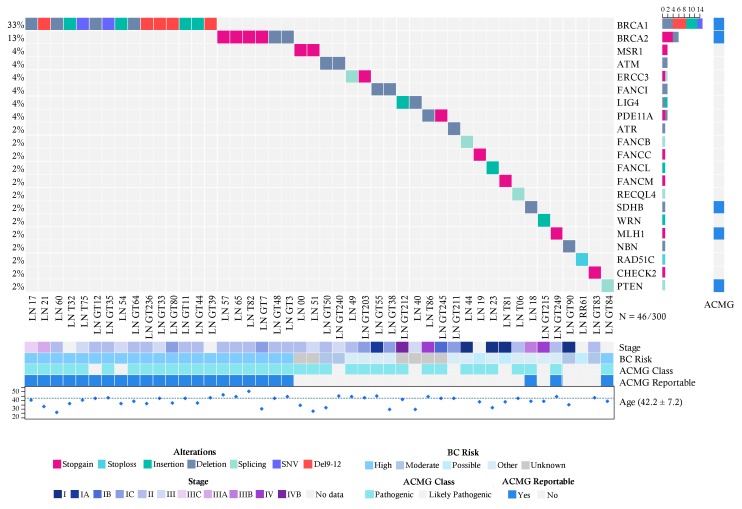
Allelic distribution of the pathogenic variants in patients with cancer. The grid panel depicts the pathogenic mutations found in each patient color-coded for each type. Right panel: gene reportable by the suggestion of the ACMG (light blue = yes, gray = no). Bottom axis: patient ID. Left axis: relative frequency of mutations per gene. Right axis: mutated gene. Right bar plot: absolute frequency and type of pathogenic mutation per gene. Bottom panel indicates: stage (I-IVB); risk associated with a pathogenic variant; ACMG variant class (pathogenic, likely pathogenic); gene reportable by the suggestion of the ACMG (light blue = yes, gray = no); age distribution.

In the group diagnosed with breast cancer (300 cases), we detected 46 pathogenic or likely pathogenic variants in 46 patients (Figure 1; Table S1), including 22 frameshift changes, 13 stop gain/loss mutations and 4 splicing variants. Fifty-six percent (26/46) of mutations detected were already reported in ClinVar as pathogenic. Twenty six percent (12/46) of the pathogenic variants were recurrent, and no genetic alteration was found in 73.6% (221/300) of the patients (Figure 1).

Notably, *BRCA1* (5%, 15/300), *BRCA2* (2%, 6/300) and *PTEN* (0.3%, 1/300) were the only high-risk mutated genes associated with HBOC (Figure 1). No Ashkenazi founder mutations were detected and no pathogenic variants were found in other high-risk HBOC genes such as *TP53*, *CDH1*, *PALB2* and *STK11*. Pathogenic alterations in moderate-risk genes were found in *ATM* (0.6%, 2/300), CHEK2 (0.3% 1/300) and NBN (0.3%) (Table S1) (Figure 1).

### 2.3. Pathogenic Variants in Familial Breast Cancer Risk Patients without Cancer Diagnosis

**Figure 2:**
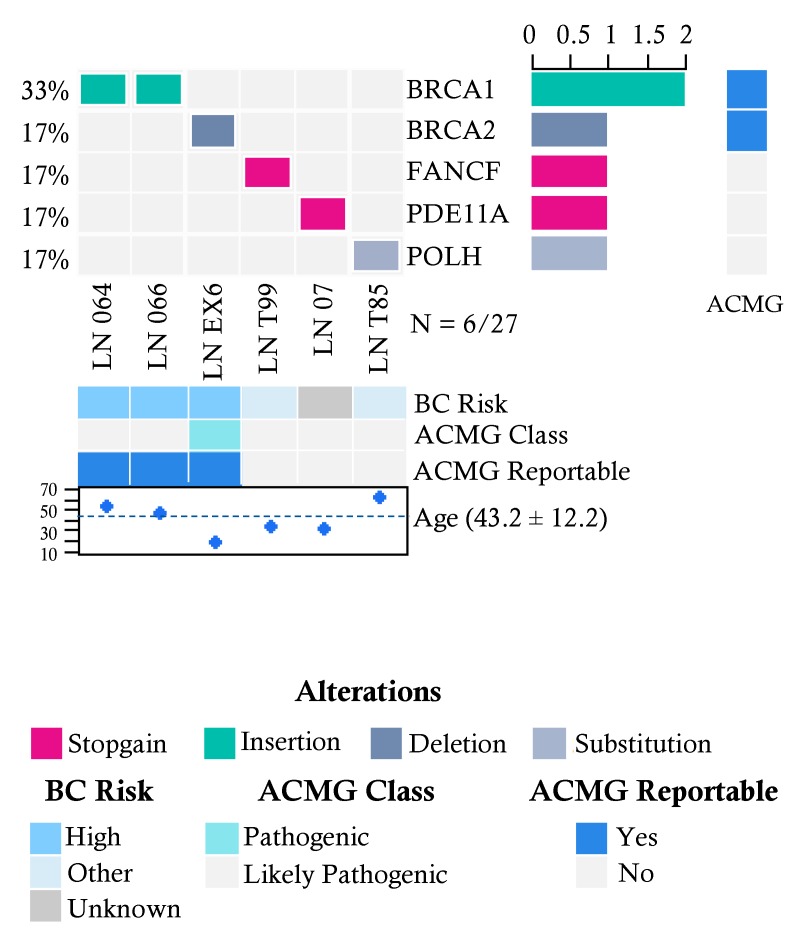
Allelic distribution of the pathogenic variants in high-risk patients with a severe family history of cancer. The grid panel depicts the pathogenic mutations found in each patient color-coded for each type. Right panel: gene reportable by the suggestion of the ACMG (light blue = yes, gray = no). Bottom axis: patient ID. Left axis: relative frequency of mutations per gene. Right axis: mutated gene. Right bar plot: absolute frequency and type of pathogenic mutation per gene. Bottom panel indicates: risk associated with a pathogenic variant; ACMG variant class (pathogenic, likely pathogenic); gene reportable by the suggestion of the ACMG (light blue = yes, gray = no); age distribution.

In the group of 27 patients without cancer and with suspicion of familial breast cancer risk, we found pathogenic variants in 6 individuals (22%) (Figure 2). The affected genes were *BRCA1* (2/27), *BRCA2* (1/27), *FANCF* (1/27), *PDE11A* (1/27) and *POLH* (1/27).

### 2.4. Recurrent Mutations in BRCA1 and BRCA2 in Both Groups

**Figure 3:**
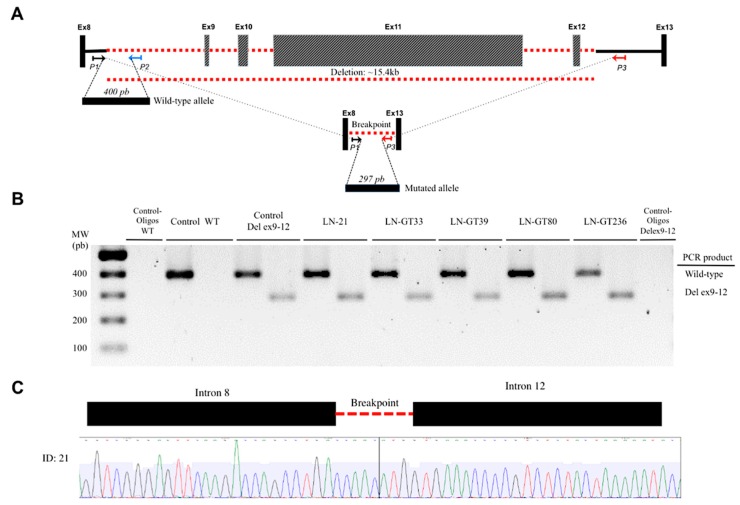
Detection of BRCA1 deletion of exons 9-12. (**A**) The locus of exons BRCA1 8 to 13 is indicated. Exons (not to scale) are depicted as boxes and introns as lines, where the discontinuous red line indicates the deleted exons 9-12. The location and orientation of the primers used for amplification of the wild-type (P1, P2) and the mutant alleles (P1, P3) are shown. The PCR products for both amplicons are depicted as horizontal boxes, with their respective number of bp. (**B**) The resolved PCR products of the patients with the deletion are shown, along with the wild-type and negative controls. (**C**) The electropherogram of the sequence shows the intron-intron junction in the deletion.

There were 2 recurrent pathogenic alleles in *BRCA1*, including p.G228fs in five individuals (29%, 5/17), and the Mexican founder mutation in *BRCA1* (deletion of exons 9-12), was present in 29% (5/17) (Figure 1, Figure 2 and Figure 3). One recurrent mutation was found in *BRCA2*: p.R2494X, which was detected in 2 patients (Table S1).

### 2.5. Pathogenic Variants in Genes with Unknown Risk in Breast Cancer

Deleterious variants in low risk HBOC genes were found in two cancer patients in the genes *FANCI* (0.6%, 2/300), *ERCC3* (0.6%), and in one patient in the genes *ATR*, *FANCB*, *FANCC*, *FANCF*, *FANCL*, *FANCM*, *MLH1*, *RAD51C*, *POLH*, *RECQL4*, *SDHB* and *WRN* (0.3%, 1/300) (Figure 1 and Figure 2). Furthermore, analysis of genes associated with risk of other inherited neoplastic syndromes different to HBOC identified heterozygous pathogenic variants for *MSR1* (4%, 2/52), *LIG4* (4%, 2/52) and *PDE11A* (6%, 3/52) in the affected women in both groups (Figure 1 and Figure 2, Table 3).

### 2.6. Description of Variants with Unknown Clinical Significance by Phosphorylation Site Disruption Analysis

We found 38 VUS in 21 genes, 4 of which were found in homozygosity (Table S2). These VUS have MAF < 0.001 in ExAC, and 1000 Genomes databases and not all of them are classified as VUS in ClinVar. To better define the potential effect of VUS in gene functionality, we evaluated the impact of the amino acid change in the context of phosphorylation sites. There was no enrichment in these sites for the occurrence of VUS. However, changes that potentially affect the phosphorylation regulation were found in the *AIP* and *APC* genes (Figure S2). The changes affected the FKBP C domain and *APC* basic domain for *AIP* and *APC*, respectively.

## 3. Discussion

In this work, we evaluated genetic alterations in an expanded panel of 143 genes associated with oncologic inherited diseases, including breast, colon, gastric, among others, by MPS in two groups of high-risk HBOC patients. This is the first study in a Latin American population that analyzes a large cancer risk gene panel by MPS. Overall in all the individuals included in this study, we detected pathogenic variants in 16% (52/327), including 7% (24/327) of variants in *BRCA1*/2, and 8% (28/327) in genes other than *BRCA1*/2 (Table 1). These mutations were found in 21 genes previously associated with more than 25 inherited conditions related to cancer (Table 3). Globally, 8% (27/327) of patients had a pathogenic mutation in one of the genes categorized by the American College of Medical Genetics ACMG as a secondary finding with clinical validity and utility to improve medical outcome [30]. Interestingly, half of the pathogenic variants, 50% (26/52), have not been reported before in any Latin-American population, which highlights the current need to expand the evaluation of the genetic diversity of under-studied, mixed populations such as Mexicans and its association to HBOC. These results also confirm the high level of locus heterogeneity that has been described for HBOC [6,23,31] (Table 1). 

Age at diagnosis was the only epidemiological or clinical variable associated with the presence of a pathogenic mutation in breast cancer cases, supporting the NCCN criteria for HBOC. Several studies have identified additional life style and genetic risk factors modifying the penetrance in *BRCA1*/2 mutation carriers [32]. A recent meta-analysis evaluated potentially risk-modification factors for *BRCA1* and *BRCA2* carriers such as age at first pregnancy, parity, breastfeeding, use of oral contraceptives, smoking and radiation exposure [32]. The loss of at least 10 pounds of body weight before the age of 30 was associated with a reduced risk of BC between 30 to 49 years in *BRCA1* mutation carriers [32]. Interestingly, in our analysis around 60% of BC cases were overweight or obese at the time of diagnosis but only 15% of patients had a pathogenic mutation in any of the 143 genes evaluated.

In addition, genetic studies of risk-modifiers focused in the *BRCA1* and *BRCA2* genes have identified 26 and 16 SNPs associated with BC risk in *BRCA1*/2 mutation carriers, which have small associated effect sizes (1.05–1.26) per copy of the minor allele [10,33,34]. Given the descriptive focus of our study, the possible combined effect of common low-risk alleles with the detected pathogenic variants was not evaluated. However, these genetic risk-modifiers are thought to account for less than 10% of the genetic variance [10,33,35]. The lack of evidence that associates these modifying factors with pathogenic variants in other genes of high- and medium-penetrance that participate in the development of HBOC is an unsolved concern. These potential allelic interactions could act as genetic modifiers of the risk of pathogenic variants present (especially) in low penetrance genes and might account for the clinical differences in disease presentation and outcome [36].

To our knowledge there is no information on modifiable risk factors for HBOC pathogenic variant carriers in Latin America available to compare the findings from our study. Larger prospective studies on HBOC mutation carriers that incorporate information on a variety of environmental exposures, ancestry and lifestyle factors are required to identify modifying risk factors in Latin America. These studies should include index patients and selected families in diverse representative populations to provide (i) reliable estimates of the allelic frequencies of the pathogenic alleles and modifying variants, (ii) the risk they confer and that may ultimately (iii) facilitate genetic counseling for patients carrying pathogenic variants with demonstrated clinical utility.

An interesting finding was that patients in the older age group (60–69 years) were characterized by presenting with early stage tumors (I/II) and absence of mutations even though they fulfilled the NCNN criteria for HBOC. Given the late presentation and the early stage of the disease, it is possible that these patients may have single pathogenic variants in genes or loci of lower risk not included in our analysis. Another possibility is that these patients may carry a combination of different low-risk loci (not identified in this work) that have additive or epistatic effects, as has been observed in other types of cancer [37,38]. Some reports have previously shown a series of low-risk alleles in genes involved in DNA repair, modification and metabolism related pathways, which act in concert to increase the risk of BC [39,40,41,42]. With the further generalized implementation of WES and WGS population-scale studies these potential multi-allelic interactions will be identified. In addition, a higher frequency of early stage tumors in patients above 60 years with a strong family history of BC might be explained by the increased awareness of this group to comply with current BC screening guidelines [43]. This represents a direct and additional benefit for early detection when identifying high-risk BC cases.

Pathogenic alterations in the HBOC moderate-risk genes *ATM*, *CHEK2* and *NBN*, were found in a frequency of 0.6%, 0.3%, and 0.3%, respectively. Additionally, we found 7 monoallelic pathogenic variants (2%, 7/327) in 6 genes (besides *BRCA1*/2) of the interstrand crosslink DNA repair Fanconi anemia pathway (*FANCB*, *FANCC*, *FANCF*, *FANCI*, *FANCL*, *FANCM*) and 1 in *RAD51C*, a Fanconi-like phenotype gene [44]. The allelic frequency of these variants in the Latin American population spans the 0–0.0015 interval (ExAC). These results confirm findings from other multi-gene panel studies in HBOC patients (Table 1). Although strong evidence regarding the contribution of mutations in some Fanconi anemia genes to HBOC is still limited [45,46], our results provide additional support for this potential association. 

Interestingly, we detected pathogenic variants in *MSR1*, *LIG4* and *PDE11A*, genes not previously associated with HBOC, both in BC patients and high-risk cases. Moreover, the mutation *MSR1* p.R293X was found in two unrelated patients. This mutation has been associated with Barrett’s esophagus and esophageal adenocarcinoma in European families [47], and with hereditary prostate cancer [48]; although contradictory results also exist [49,50]. The mutation p.R505fs in the Non-homologous end joining (NHEJ) ligase *LIG4*, found in one patient, has an allele frequency of 0.0000247 in ExAC. This mutation is located in the ATP dependent DNA ligase C terminal region, and produces a protein lacking both of BRCT-I and BRCT-II domains that are required for chromatin binding [51], abrogating *LIG4* function. Recently, germline mutations in *LIG4* have been suggested to predispose to diffuse large B-cell lymphomas [52] and to sensitize cell to ionizing radiation, causing immunodeficiency and delay in growth and development in homozygous or compound heterozygous carriers (OMIM#606593) [53]. Given the biochemical function of *LIG4* in NHEJ and the low prevalence of its mutations, germline monoallelic mutations could influence HBOC risk, although additional studies are needed to establish this association. In one patient we found a frameshift pathogenic variant p.G57fs in *PDE11A*, a gene previously associated with different neoplasms including Carney multiple neoplasia complex, prostate cancer and testicular germ cell tumors [54,55].

Overall, 10.8% of patients that were negative for a pathogenic mutation in any of the 143 genes tested had VUS defined following the ACMG criteria. VUS constitute a universal concern in cancer genetics diagnostic settings. The risk conferred by VUS must be addressed by generating more evidence of their allelic frequency in different populations, and by conducting co-segregation analyses, as well as efforts to define their function at protein level using experimental models. Remarkably, we found 2 VUS-*AIP* p.V49M in homozygosis and *APC* p.S2535G in heterozygosis—that potentially affect the phosphorylation regulation of the protein. In fact, mutations in the chaperone aryl hydrocarbon receptor-interacting protein (AIP) have been found in familial cases of pituitary adenomas [56]. Experimental in vitro evidence showed that AIP V49M interferes with AIP activity and stability [57]. *APC* p.S2535G was predicted to disrupt a phosphorylation site in the protein basic domain, which interacts with the microtubules [58]. Neither this amino acid change nor any other change in this position has been reported in COSMIC, OMIM or ClinVar. Consequently, further functional studies are needed to determine the impact of the *APC* p.S2535G variant. 

On the other hand, seventy-four percent of all patients did not harbor alterations in any of the 143 genes studied. Even though we tested the deletion of exons 9-12 in *BRCA1*, a mutation with a founder effect and the highest frequency reported in Mexican population [59], additional larger rearrangements, undetectable by MPS could account for the lack of mutation detection in this group of patients. The frequency of large genomic rearrangements in *BRCA1*/2 varies considerably among populations but higher frequencies are related to founder effect variants [60]. In our study, we found that almost one out of three patients (5/17) with *BRCA1* pathogenic variants had the Mexican founder mutation (deletion of exons 9-12), which highlights the additional value of evaluating this alteration through a rapid test, such as the one we used. It is also possible that patients who tested negative for any of the genes evaluated may harbor variants in noncoding regions that we did not analyzed. Additional mechanisms of pathogenesis that may play a role in susceptibility to BC might include pathogenic variants affecting splicing mechanisms that disrupt RNA-binding protein (RBBSs) and splicing regulatory (SRBSs) binding sites as well as transcription factor binding site disruption or promoter mutations [31,61]. 

An additional limitation of this study is the lack of population paired-controls, which could lead to the wrong attribution of common, non-deleterious variants as pathogenic, and which also could eliminate an important amount of common VUS present in this population. It has been described that each person carries up to 100 loss-of-function variants, thirty of which could be in homozygosis, and these are not necessarily disease-causing variants [62,63]. To exclude for non-pathogenic natural variation, we used the largest international databases (ExAC, 1000G, ESP) available in our filtering algorithm. These repositories contain whole genome and exome information from a large number (N = 60,706) of sequenced individuals, with broad ancestral diversity, including 5789 Latinos (2254 males and 3535 females). It has been reported that ExAC is not overrepresented for pathogenic variants, which supports its use to estimate normal variation [64]. We estimate that this strategy may have helped to palliate the effect of lack of paired-controls on our study.

Future germline analyses of cohort studies and population based case-control studies specifically focused on underrepresented populations such as the Latin American region and including women with BC with and without HBOC susceptibility are necessary to validate our results.

Overall, we found 54% of pathogenic variants in genes other than *BRCA1* and *BRCA2*. Consistently, other studies using a panel sequencing approach have found a proportion of 5–64% of pathogenic variants in non-*BRCA* genes (Table 1). The rate of pathogenic variant detection in these studies tend to be dependent on the total number of genes analyzed, rather than the number of individuals studied. For example, the largest study evaluated a panel of 21 genes in 65,057 patients with BC and found pathogenic variants in 8 non-*BRCA* genes [10], and our study using a panel of 143 genes in 327 individuals, detected pathogenic variants in 21 non-*BRCA* cancer-associated genes. Therefore, to further elucidate the wider variation in genes with pathogenic variants that influence HBOC, more studies that investigate larger panels, or ultimately the whole exomes or genomes are needed. Likewise, penetrance and polygenic analyses of rare and common variation will aid to provide more accurate assessment of genetic cancer risk in the clinical setting.

The findings of this work have relevant clinical and public health implications. The frequency of mutations we found in high and intermediate penetrance HBOC associated genes, emphasizes the additional advantage of using a complete gene panel testing instead of single selected mutation approach, increasing the number of identified high-risk individuals who might benefit from personalized prevention or clinical intervention programs. In addition, the implementation of extended gene panels constitutes an efficient development to accelerate de detection of a broader number of high-risk mutation carriers. We detected patients with mutations in *BRCA1* and *BRCA2* but also in the *MLH1*, *SDHB* and *PTEN* genes that are suggested to be reported by the ACMG given their risk to develop other hereditary conditions [30]. The gradual adoption of clinically informative gene panel testing along with genetic counseling programs will eventually be a key component in the prevention of cancer and other genetic diseases. Lastly, our results highlight the current necessity for the establishment of prospective cohorts in understudied populations, such as the Latin American, to better establish factors that modify penetrance and to identify association relationships of new genetic variants with the disease. These studies could provide enough evidence to direct public health guidelines for risk assessment programs in specific populations including genetic testing recommendations, lifestyle and treatment interventions.

## 4. Materials and Methods

### 4.1. Study Population and Data Collection

A total of 327 patients were enrolled based on criteria established in the Genetic/Familial High-Risk Assessment: Breast and Ovarian of the National Comprehensive Cancer Network (NCCN) guidelines, version 2.2015 (https://www.nccn.org/). A transversal series of 300 Mexican female patients diagnosed with primary breast cancer (stages I–IV) were recruited at 4 centers from different states in Mexico: Instituto de Salud del Estado de México, Instituto Estatal de Cancerología de Guerrero, Centro de Investigación Biomédica, Torreón, Coahuila and Hospital de Oncología del CMN Siglo XXI de la Ciudad de México. A second group of 27 individuals without cancer diagnosis who meet NCCN criteria for HBOC susceptibility were additionally included. The protocol was approved by the Ethics Committee of each center (IECG-CEICANCL290515-05GENCMAHER; IECC-2015-01; ISEM-02092015; INSP-CI:1065; INSP-341) and was conducted in accordance with the Declaration of Helsinki. Patients provided written informed consent for participation in this study, and their samples were anonymized and sent to the Laboratorio Nacional en Salud: Diagnóstico Molecular y Efecto Ambiental en Enfermedades Crónico-Degenerativas, Facultad de Estudios Superiores Iztacala, UNAM. Epidemiological and clinical information was obtained from hospital records when available. After the review of their clinical records and age of onset (<45 years), all patients with suspicion of HBOC were invited to participate in this study. After a complete and detailed explanation of the study and written informed consent, a questionnaire of enrollment was used to evaluate the fulfillment of inclusion criteria.

### 4.2. Sample Preparation and DNA Extraction

For all patients enrolled, 4 mL samples of blood were collected and stored locally at −80 °C. The period between sample collection and freezing never exceeded 36 h. Peripheral blood DNA was extracted with the DNeasy Blood & Tissue Kit (Qiagen, Hilden, Germany) following manufacturer’s instructions. DNA concentration was quantified with the Qubit dsDNA HS Assay Kit (Invitrogen, Carlsbad, USA) and the integrity and purity of the material was verified by agarose gel electrophoresis and spectrophotometry, respectively. 

### 4.3. Library Preparation and Massive Parallel Sequencing

Peripheral Blood DNA was used for library preparation with the GeneRead Cancer Predisposition V2 Kit (Qiagen, Hilden, Germany), which targets 143 genes, which loss of function is a well-known mechanism associated with 88 inherited oncologic diseases based on data from the College of American Pathologists (CAP) guidelines, NCCN guidelines, late-stage clinical trials, The Cancer Genome Atlas (TCGA), and Ingenuity^®^ Knowledge Base. The amplification was divided in 4-pool PCR reactions with a total of 6582 amplicons. Pair-end sequencing was performed with the MiSeq System platform (Illumina, San Diego, USA). Briefly, 40 ± 2.5 ng of DNA was amplified with the GeneRead DNAseq Gene Panel Kit (Qiagen, Hilden, Germany) and purified with Agencourt AMPure XP magnetic Beads (Beckman Coulter, Brea, USA). The amplified fragments were end-repaired, dA-tailed and the adapter GeneRead Adapter 1 Set plex (Qiagen, Hilden, Germany) was ligated using the GeneRead DNA Library I Core Kit. Amplified segments were then size-selected (200–300 bp) using Agencourt AMPure XP magnetic beads (Beckman Coulter, Brea, USA). New England Biolabs (Ipswich, USA) barcodes were incorporated by PCR amplification in 10 PCR cycles and the products were purified. The libraries were diluted to 4.0 nM and were pooled in batches of 60-80 samples. Library quality was evaluated by DNA quantification with Qubit after size-selection, and by Bioanalyzer (Agilent, Santa Clara, USA) profiling with the High Sensitivity DNA Kit after adaptor-ligated molecules amplification and final library pooling. Pooled barcoded libraries were diluted to 15.0 pM and sequenced with a MiSeq Reagent Kit V2 2 × 150 cycles (Illumina, San Diego, USA) to reach a theoretical average coverage of 100× for each sample. 

### 4.4. Pathogenic Variant Detection

Alignment and variant calling were performed with BWA and GATK (Broad Institute, Cambridge, USA). FastQC files were aligned to the human genome reference hg19 with BWA-MEM; indels were realigned and bases recalibrated. Adaptors were soft-clipped and reads with <20 bp were eliminated. The overall (327) mean sequencing depth of all samples was 70.3× (SD: 21.35) with a range 30–156×, excluding one sample with depth 20× (Figure S1). Variant calling was done with HaplotypeCaller (Broad Institute, Cambridge, USA). Variants were annotated with ANNOVAR and InterVar [65,66]. Mutation description follows Human Genome Variation Society (HGVS) nomenclature (http://www.hgvs.org/). Variant classification followed the five-tier criteria of the American College of Medical Genetics and Genomics (ACMG) [29] and was manually curated. We excluded variants that were synonymous, with depth <5.0× or with mutant allele fraction <20% and those present in homopolymeric tracts >8 bp. All splicing and null variants (stop-gain/loss, frameshift indels) and missense variants defined as pathogenic in ClinVar were considered unequivocally pathogenic (https://www.ncbi.nlm.nih.gov/clinvar). Null variants present at the 3′ extreme end of the gene that were reported as conflicting in ClinVar were classified as unknown clinical significance (VUS). Minor allelic frequency <0.001 in either the ExAC database, 1000 Genomes (1000G) project or the Exome Sequencing Project (ESP6500) was used to capture rare, potentially pathogenic null and missense variants. Low frequency (<0.001) missense variants predicted as deleterious alleles by SIFT or PolyPhen-2 but with no further evidence of pathogenicity in vitro/vivo or clinically were classified as VUS. All filtered variants were manually curated by inspection of the BAM files with the IGV software (Broad Institute). All pathogenic variants were confirmed by two independent assays of Sanger sequencing. Variants in *BRCA1* and *BRCA2* were further assessed in the Huntsman Cancer Institute Breast Cancer Genes Prior Probabilities site (http://priors.hci.utah.edu/PRIORS/index.php) to evaluate their potential impact. Variants in *MLH1*, *MSH2*, *MSH6*, *PMS2* were also investigated in the Leiden Open Variation Database (http://hci-lovd.hci.utah.edu/home.php).

### 4.5. Detection of Exon 9-12 Deletion in BRCA1

The deletion in exons 9-12 founder mutation was detected by PCR amplification of the mutant and wildtype allele, using specific primers based on the Weitzel et al. method [59]. The PCR products were resolved in 1.5% agarose gels to identify the amplification of the truncated allele and sequenced.

### 4.6. Phosphorylation Site Disruption Analysis

To evaluate the impact of missense changes in phosphorylation sites, the protein sequences and amino acid changes of all VUS variants (Table S2) were submitted to the ReKINect portal (http://rekinect.science/home). Only mutations predicted to disrupt the phosphorylation site and those which have previous evidence of functional impact in experimental studies were considered.

### 4.7. Statistical Analyses

Characteristics of cases with confirmed diagnosis of breast cancer were summarized with descriptive statistics. The association between demographic and clinical characteristics on the presence of pathological mutations was assessed using univariate analyses (unadjusted logistic regression model). Age at diagnosis and BMI were included as continuous variables, whereas all other factors were considered to be categorical variables. The logistic regression model utilized all available data (complete and missing). *p* values of less than 0.05 were considered to indicate statistical significance. All the analyses were conducted using STATA 13.0.

## 5. Conclusions

Our results show that 16% of the patients with suspicion of HBOC carried a pathogenic mutation in at least one of the 143 genes tested. Fifty-four percent of all pathogenic alterations were not present in *BRCA1* and *BRCA2*, highlighting the locus heterogeneity of this disease. We found 10% of patients with VUS, which require further studies to establish their significance. The genetic information derived from this study could guide the treatment, appropriate follow-up and prophylactic measures in these families and our findings emphasize the benefit of gene panel sequencing service for candidate patients. Although currently clinical guidelines for patients with the pathogenic mutations detected in several of these genes are lacking, the detection of these variants together with a suggestive family history may warrant for a post-test management change, including close follow-up and monitoring. Future efforts will collectively provide enough evidence of the clinical impact of these variants and will foster the development of consensus population-specific guidelines for clinical management.

## Figures and Tables

**Table 1:** Summary of gene panel studies in hereditary breast cancer.

Year	Genes	Sample	Country	Methods	*BRCA* Frequency ^1^	Non*-BRCA* Frequency ^3^	Non*–BRCA* Genes	Ref.
					Proportion ^2^	Proportion ^4^		
2018	143	327	Mexico	GeneRead (Qiagen)	7.3% (24/327)	8.5% (28/327)	*MSR1*, *ATM*, *ERCC3*, *FANCI*, *LIG4*, *PDE11A*, *ATR*, *FANCB*, *FANCC*, *FANCL*, *FANCM*, *RECQL4*, *SDHB*, *WRN*, *MLH1*, *NBN*, *RAD51C*, *CHEK2*, *FANCF*, *POLH* and *PTEN*	This study
					46.1% (24/52)	53.8% (28/52)		
2018	35	120	Korea	OncoRisk (Celemics)	Negative	7.5% (9/120)	*TP53*, *PALB2*, *BARD1* and *MRE11A*	[9]
2017	21	65,057	USA Multicentric	Multiple	2.8% (1874/65057)	5.3% (3422/65057)	*CDH1*, *PTEN*, *TP53*, *ATM*, *BARD1*, *CHEK2*, *PALB2*, and *RAD51D*.	[10]
					35% (1874/5296)	64% (3422/5296)		
2017	10	581	Germany	TruSight Cancer	12.4% (72/581)	5.5% (32/581)	*CHEK2*, *PALB2*, *NBN*, *RAD51C*, *ATM*, *TP53*, *RAD51D* and *MSH6*	[11]
					69% (72/104)	30% (32/104)		
2017	16	453	Palestine	SureSelect (Agilent)	6.8% (31/453)	6.6 (30/453)	*TP53* (founder mutation), *ATM*, *CHEK2*, *BARD1*, *BRIP1*, *PALB2*, *MRE11A*, *PTEN*, and *XRCC2*	[12]
					50.8% (31/61)	49.1% (30/61)		
2017	94	255	Italy	Trusight Cancer (Illumina)	22.3% (57/255)	6.6% (17/255)	*PALB2*, *ATM*, *BRIP1*, *RAD51D*, *MSH6*, *PPM1D*, *RECQL4*, *ERCC3*, *TSC2*, *SLX4* and other Fanconi anemia genes	[13]
					77% (57/74)	22.9% (17/74)		
2017	27	240 120 = BC 120 = High-risk	China	BGI chip (Blackbird platform)	5.8% (14/240)	9.6% (23/240)	*MUTYH*, *CHEK2*, *PALB2*, *ATM*, *BARD1*, *NBN*, *RAD51C*, *TP53* and *BRIP1*	[14]
					38% (14/37)	62% (23/37)		
2017	25	85	Colombia	MyRisk (Myriad)	17.6% (15/85)	4.7% (4/85)	*PALB2*, *ATM*, *MSH2* and *PMS2*	[15]
					79% (15/19)	21% (4/19)		
2016	29	10,030	USA	SureSelect targeted capture	2.54% (255/10,030)	6.7% (682/10,030)	*MLH1*, *MSH2*, *MSH6*, *PMS2*, *EPCAM*, *APC*, *MUTYH*, *CDH1*, *PTEN*, *STK11*, and *TP53*	[16]
					27% (255/937)	73% (682/937)		
2016	4	1427 479 = Sanger 948 = NGS	China	PCR design	8.8% (126/1427)	0.49% (7/1427)	*TP53* and *PTEN*	[17]
					95% (126/133)	5% (7/133)		
2016	19	684 BRCA negative patients	Australia	Agilent Target Enrichment	Negative	11.1% (76/684)	*TP53*, *PALB2*, *ATM*, *CHEK2*, *CDH1*, *PTEN* and *STK11* Segregation study: *CDH1*, *CHEK2*, *PALB2* and *TP53*	[18]
2016	13	141	India	Trusight Cancer	4.9% (7/141)	9.9% (14/141)	*ATM*, *BRIP1*, *CHEK2*, *PALB2*, *RAD51C* and *TP53*	[19]
					33% (7/21)	66% (14/21)		
2016	68	133	Taiwan	NimblGen capture (Roche)	15% (20/133)	7.5% (10/133)	*RAD50*, *TP53*, *ATM*, *BRIP1*, *FANCI*, *MSH2*, *MUTYH*, and *RAD51C*	[20]
					66% (20/30)	33% (10/30)		
2015	25	2158 Cohort 1 = 1781 (*BRCA1*/2) Cohort 2 = 377 negative BRCA)	USA	RainDance Thunderstorm emulsion polymerase chain reaction (PCR) system	Cohort 1 9.3% (165/1781) Cohort 2 NA	Cohort 1 4.2% (15/377) Cohort 2 3.7% (14/377)	*CHEK2*, *ATM* and *PALB2*	[21]
2015	29	Total: 1062 735-clinically representative	USA	SureSelect and Integrated DNA Technologies	9% (66/735)	3.9% (26/735)	*ATM*, *PALB2*, *CHEK2*, *MLH1*, *MSH2*, *MSH6*, and *PMS2*	[22]
					72% (66/92)	28% (26/92)		
2015	29 (Invitae) 25 (Myriad)	1046 BRCA negative patients	USA	Hereditary Cancer Syndromes test (Invitae) MyRisk test (Myriad Genetics)	Negative	3.8% (40/1046)	*CHEK2*, *ATM*, *PALB2*	[23]
2015	94 genes and 284 SNPs	620	Germany	TruSight (Illumina) and Haloplex	9.2% (57/620)	2.9% (18/620)	*CHEK2*, *ATM*, *CDH1*, *NBN*, *PALB2* and *TP53*	[24]
					76% (57/75)	24% (18/75)		
2015	25	155	Japan	AmpliSeq Library Kit 2.0	7% (11/155)	1.9% (3/155)	*ATM*, *MRE11A* and *MSH6*	[25]
					78.5% (11/14)	21.5% (3/14)		

^1^ Absolute frequency of patients with a pathogenic variant in *BRCA1* and *BRCA2*. ^2^ Proportion of pathogenic variants in *BRCA1* and *BRCA2* relative to other genes. ^3^ Absolute frequency of patients with a pathogenic variant in non-*BRCA* cancer-associated genes. ^4^ Proportion of pathogenic variants in non-*BRCA* cancer-associated genes relative to BRCA1 and BRCA2.

**Table 2:** Clinical and epidemiological characteristics of 300 women with breast cancer and 27 familial breast cancer risk women.

Epidemiological and Clinical Characteristics	n	(%)
	300	(100)
**Age**		
<40 years	125	(41.7)
41–50 years	135	(45.0)
>50 years	24	(8.0)
Missing	16	(5.3)
**BMI**		
Underweight (<18.5)	1	(0.3)
Normal weight (18.5 < 25)	107	(35.7)
Overweight (25.0 < 30)	118	(39.3)
Obese (30.0 < 40)	66	(22.0)
Extreme obese (>40)	3	(1.0)
Missing	5	(1.7)
**Current Alcohol Drinker**		
No	278	(92.7)
Yes	16	(5.3)
Missing	6	(2.0)
**Current Tobacco Smoker**		
No	84	(28.0)
Yes	74	(24.7)
Missing	142	(47.3)
**Pregnancy**		
Yes	256	(85.4)
No	43	(14.3)
Missing	1	(0.3)
**Ever Use of Oral Contraceptives**		
Yes	115	(38.3)
No	179	(59.7)
Missing	6	(2.0)
**Family History of Cancer**		
Yes	214	(71.3)
No	80	(26.7)
Missing	6	(2.0)
**Histopathological Subtype**		
DCIS	43	(14.4)
LCIS	18	(6.0)
IDC	189	(63.0)
ILC	16	(5.3)
MC	3	(1.0)
Missing	31	(10.3)
**Stage**		
I	51	(17.0)
II	115	(38.3)
III	86	(28.7)
IV	10	(3.3)
Missing	38	(12.7)
**ER Status**		
Negative	38	(12.7)
Positive	20	(6.6)
Missing	242	(80.7)
**PR Status**		
Negative	135	(45.0)
Positive	22	(7.3)
Missing	143	(47.7)
**HER2 Status**		
Negative	7	(2.3)
Positive	45	(15.0)
Missing	248	(82.7)
Mutational Status *		
Non-mutated	254	(84.7)
Mutated	46	(15.3)

DCIS = Ductal carcinoma in situ, LCIS = Lobular carcinoma in situ, IDC = Invasive ductal carcinoma, ILC = Invasive lobular carcinoma, MC = Medullary carcinoma. ER = Estrogen receptor, PR = Progesterone receptor. * Mutational status is based on the presence of a pathogenic mutation in any of the 143 genes analyzed.

**Table 3:** Syndromes associated with the pathogenic variants detected.

Gene	Frequency	Syndromes (OMIM)	Breast Cancer Risk	Inherited Pattern	Signaling Pathways	Reportable in ACMG *
*BRCA1*	17	Hereditary Breast and Ovarian Cancer	High	AD	Double strand damage (HR)	Yes
*BRCA2*	11	Fanconi Anemia/Hereditary Breast and Ovarian Cancer/Familiar Pancreatic Cancer/Hereditary Prostate Cancer	High	AD/AR	Double strand damage (HR)	Yes
*PDE11A*	3	Pigmented nodular adrenocortical disease	Novel	AD	Catalyze the hydrolysis of cAMP and cGMP, Metabolism of purines	No
*ATM*	2	Susceptibility to breast cancer/Ataxia Telangiectasia	Moderate	AD/AR	Double strand damage (HR)	No
*ERCC3*	2	Xeroderma Pigmentosum	Not established	AR	Transcription initiation of RNA Pol II	No
*FANCI*	2	Fanconi Anemia	Not established	AR	Anemia Fanconi Pathway and Double strand damage response	No
*LIG4*	2	LIG4 Syndrome	Novel	AR	Nucleotide excision DNA repair	No
*MSR1*	2	Hereditary Barret Esophagus/Esophagus carcinoma/Hereditary prostate cancer	Novel	AD	Vesicle-mediated transport and AGE/RAGE pathway	No
*ATR*	1	Cutaneous telangiectasia and familial cancer syndrome/Seckel syndrome 1	Not established	AD/AR	Cell cycle checkpoint regulator	No
*CHEK2*	1	Li-Fraumeni syndrome/Susceptibility to breast, colorectal and prostate cancer	Moderate	AD	Cell cycle checkpoint regulator	No
*FANCB*	1	Fanconi Anemia	Not established	XLR	Anemia Fanconi Pathway and Double strand damage response	No
*FANCC*	1	Fanconi Anemia	Not established	AD/AR	Anemia Fanconi Pathway	No
*FANCF*	1	Fanconi Anemia	Not established	AR	Anemia Fanconi Pathway	No
*FANCL*	1	Fanconi Anemia	Not established	AR	Anemia Fanconi Pathway, DNA damage, Cell cycle checkpoint regulator	No
*FANCM*	1	Fanconi Anemia	Not established	AD/AR	Anemia Fanconi Pathway, Double strand DNA damage	No
*MLH1*	1	Hereditary nonpolyposis colorectal cancer, type 2/Mismatch repair cancer syndrome/Muir-Torre syndrome	Not established	AD/AR	Mismatch repair system	Yes
*NBN*	1	Aplastic Anemia/Acute lymphoblastic Leukemia/Nijmegen breakage syndrome	Moderate	AD/AR	Double strand damage respond in DNA	No
*POLH*	1	Xeroderma pigmentosum	Not established	AR	Homologous DNA recombination and strand interchange	No
*PTEN*	1	Bannayan-Riley-Ruvalcaba syndrome/Cowden syndrome	High risk	AD	Antagonizes the PI3K signaling pathway and negatively regulates the MAPK pathway	Yes
*RAD51C*	1	Fanconi Anemia/Susceptibility to breast and ovarian cancer	Not established	AD/AR	Double strand damage (HR)	No
*RECQL4*	1	Rothmund-Thompson Syndrome	Not established	AR	DNA Damage response	No
*SDHB*	1	Carney-Stratakis Syndrome	Not established	AD	Metabolism (Krebs Cycle)	Yes
*WRN*	1	Werner Syndrome	Not established	AR	C strand synthesis in telomere and cell cycle checkpoint	No

* From reference [30].

## Supplementary Materials

The following are available online at http://www.mdpi.com/2072-6694/10/10/361/s1, Figure S1: Mean sequence depth of all samples analyzed; Figure S2: Phosphorylation site disruption in AIP and APC; Table S1: Pathogenic genetic alterations detected in 327 patients; Table S2: Variants with unknown clinical significance detected in 327 patients.
