# Supplementary material for: Comprehensive Analysis of Germline Variants in Mexican Patients with Hereditary Breast and Ovarian Cancer Susceptibility
Source: Cancers (Basel). 2018 Sep 27;10(10):361. doi: 10.3390/cancers10100361 (PMC6211045; doi:10.3390/cancers10100361)
Supplement: Supplementary file 1 [file cancers-10-00361-s001.pdf]

Supplementary Materials: Comprehensive analysis of germline variants in Mexican patients with hereditary breast and ovarian cancer susceptibility

Rosalía Quezada Urban, Clara Estela Díaz Velásquez, Rina Gitler, María Patricia Rojo Castillo, Max Sirota Toporek, Andrea Figueroa Morales, Oscar Moreno García, Lizbeth García Esquivel, Gabriela Torres Mejía, Michael Dean, Iván Delgado Enciso, Héctor Ochoa Díaz López, Fernando Rodríguez León, Virginia Jan, Víctor Hugo Garzón Barrientos, Pablo Ruiz Flores, Perla Karina Espino Silva, Jorge Haro Santa Cruz, Héctor Martínez Gregorio, Ernesto Arturo Rojas Jiménez, Luis Enrique Romero Cruz, Claudia Fabiola Méndez Catalá, Rosa María Álvarez Gómez, Verónica Fragoso Ontiveros, Luis Alonso Herrera, Isabelle Romieu, Luis Ignacio Terrazas, Yolanda Irasema Chirino, Cecilia Frecha, Javier Oliver, Sandra Perdomo, Felipe Vaca Paniagua

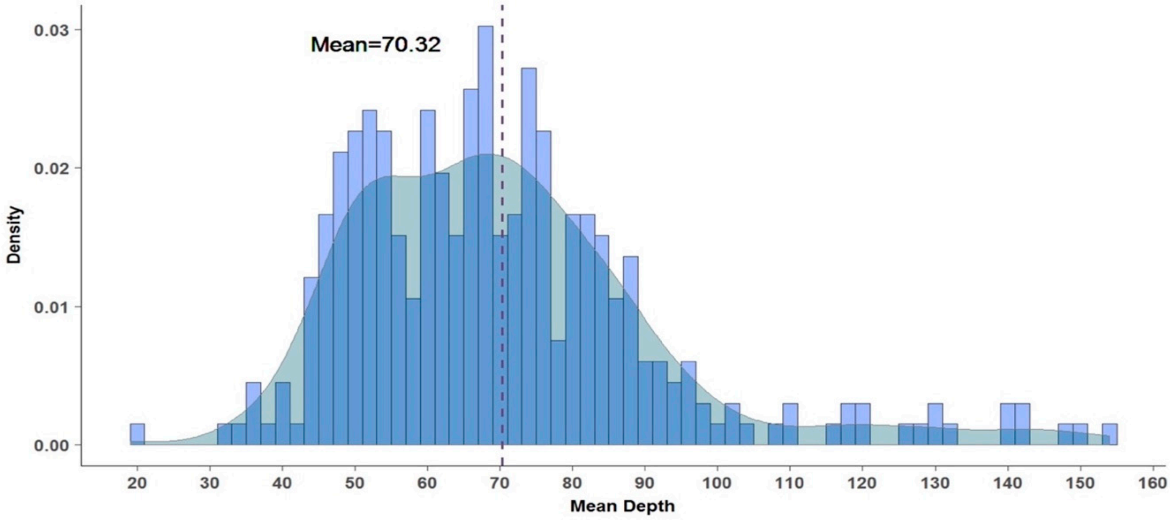

Figure S1. Mean sequence depth of all samples analyzed. Histogram of sequence depth (X) with density of all sequenced samples. Mean depth is indicated. N = 327.

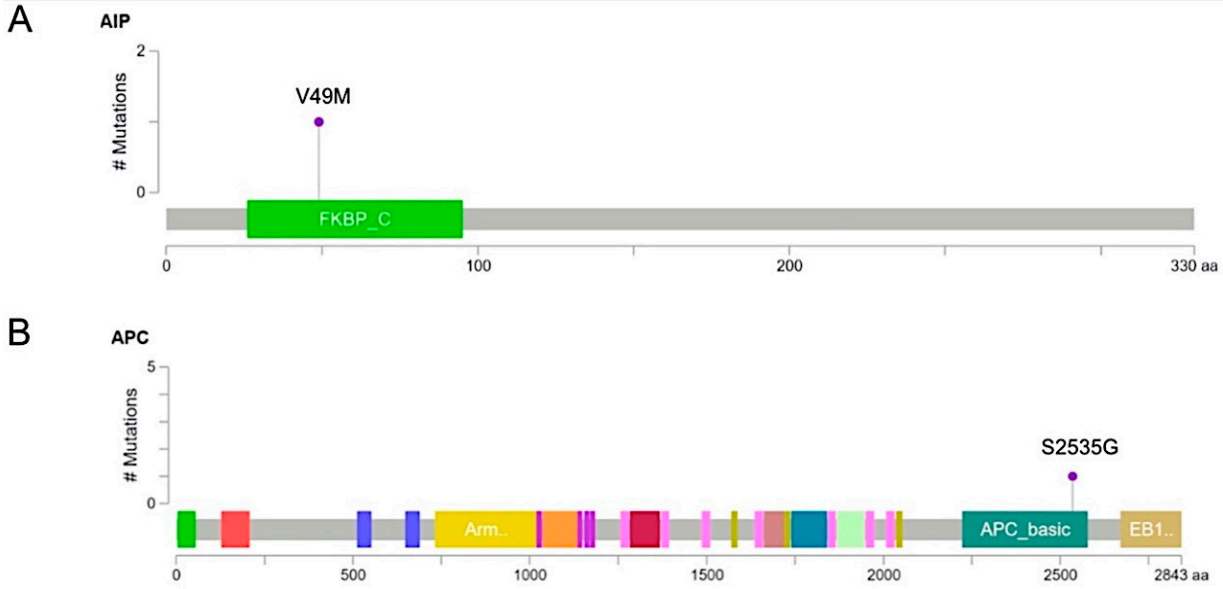

C

GENE/Mut	ReKINect	PFAM/InterPro database	Evicence in Patients/models with this change	Reference
AIP:p.V49M	Mutation around phosphorylation site	IFKBP_C domain have prolyl isomerase activity.	This specific mutation has not been detected in patients with breast cancer, but was detected in a patient with gigantism, at the germline level . This mutation was cloned and transfected in the GH3 cell line, and caused a significant loss of protein function and stability. The protein-protein interaction with PDE4A5 was similar to the WT. However V49 does not influence protein interacions because it is in the amino-terminal domain and the protein-protein interaction domain its located in the carboxy-terminal domain.	Iwata *et. al.* 2007. Formosa and Vassallo, 2017. Igreja *et al.* 2010.
APC:p.S2535G	Destruction of phosphorylation site	APC basic domain contains a high proportion of positively charged amino acids and interacts with microtubules.	No patients reported.	-

Figure S2 Phosphorylation site disruption in AIP and APC. Lollypop diagrams of AIP (A) and APC (B) display the amino acid changes predicted to affect phosphorylation sites and their positions are shown. The protein domains are illustrated as color boxes. (C) Evidence of the impact of these missense changes in silico, in models. and in patients.

Table S1. Pathogenic genetic alterations detected in 327 patients.

ID	Gene	Region	Type of change	Transcript	Exon	cDNA change	Protein change	Zygocity	Novel variant*
0	*MSR1*	exonic	stopgain	NM_002445	6	c.C877T	p.R293X	Heterozygous	No
07	*PDE11A*	exonic	stopgain	NM_016953	2	c.C985T	p.R329X	Heterozygous	No
17	*BRCA1*	Exonic	frameshift deletion	NM_007297	9	c.1719delT	p.I573fs	Heterozygous	No
18	*SDHB*	exonic	frameshift deletion	NM_003000	2	c.166_170del	p.P56fs	Heterozygous	Yes
19	*FANCC*	exonic	stopgain	NM_000136	7	c.G673T	p.E225X	Heterozygous	No
21	*BRCA1*	exonic-intronic	larger deletion	NM_007297	9-12	-	-	Heterozygous	No
23	*FANCL*	exonic	frameshift insertion	NM_001114636	14	c.1114_1115insATTA	p.T372fs	Heterozygous	No
40	*LIG4*	exonic	frameshift deletion	NM_001098268	2	c.613delT	p.S205fs	Heterozygous	No
44	*FANCB*	splicing	splicing	NM_001018113	5	c.1105-2->TATT	-	Heterozygous	No
49	*ERCC3*	splicing	splicing	NM_000122	6	c.657+1G>A	-	Heterozygous	No
51	*MSR1*	exonic	stopgain	NM_002445	6	c.C877T	p.R293X	Heterozygous	No
54	*BRCA1*	exonic	frameshift insertion	NM_007297	9	c.683_684insAGCCATG TGG	p.G228fs	Heterozygous	Yes
57	*BRCA2*	exonic	stopgain	NM_000059	15	c.C7480T	p.R2494X	Heterozygous	No
60	*BRCA1*	exonic	frameshift deletion	NM_007297	22	c.5416delT	p.Y1806fs	Heterozygous	Yes
64	*BRCA1*	exonic	frameshift insertion	NM_007297	9	c.683_684insAGCCATG TGG	p.G228fs	Heterozygous	Yes
65	*BRCA2*	exonic	stopgain	NM_000059	15	c.C7480T	p.R2494X	Heterozygous	No
66	*BRCA1*	exonic	frameshift insertion	NM_007297	9	c.683_684insAGCCATG TGG	p.G228fs	Heterozygous	Yes
EX6	*BRCA2*	exonic	frameshift deletion	NM_000059	11	c.5112_5115del	p.R1704fs	Heterozygous	No

GT11	*BRCA1*	exonic	frameshift insertion	NM_007297	9	c.683_684insAGCCATG TGG	p.G228fs	Heterozygous	No
GT12	*BRCA1*	exonic	frameshift deletion	NM_007297	19	c.5182delA	p.M1728fs	Heterozygous	Yes
GT203	*ERCC3*	exonic	stopgain	NM_000122	8	c.C1129T	p.Q377X	Heterozygous	No
GT211	*ATR*	exonic	frameshift deletion	NM_001184	21	c.3889delG	p.V1297fs	Heterozygous	No
GT212	*LIG4*	exonic	frameshift insertion	NM_001098268	2	c.1513_1514insTC	p.R505fs	Heterozygous	No
GT215	*WRN*	exonic	frameshift insertion	NM_000553	9	c.896dupT	p.I299fs	Heterozygous	No
GT236	*BRCA1*	exonic-intronic	larger deletion	NM_007297	9-12	-	-	Heterozygous	No
GT240	*ATM*	exonic	frameshift deletion	NM_000051	37	c.5648_5655del	p.S1883fs	Heterozygous	Yes
GT245	*PDE11A*	exonic	stopgain	NM_016953	2	c.C919T	p.R307X	Heterozygous	No
GT249	*MLH1*	exonic	stopgain	NM_000249	8	c.C676T	p.R226X	Heterozygous	No
GT3	*BRCA2*	exonic	frameshift deletion	NM_007297	14	c.4701delA	p.P1567fs	Heterozygous	No
GT33	*BRCA1*	exonic-intronic	larger deletion	NM_007297	9-12	-	-	Heterozygous	No
GT35	*BRCA1*	exonic	nonsynonymous SNV	NM_007297	3	c.A70G	p.R24G	Heterozygous	No
GT38	*FANCI*	exonic	frameshift deletion	NM_018193	31	c.3312delG	p.K1104fs	Heterozygous	No
GT39	*BRCA1*	exonic-intronic	larger deletion	NM_007297	9-12	-	-	Heterozygous	No
GT44	*BRCA1*	exonic	frameshift insertion	NM_007297	9	c.683_684insAGCCATG TGG	p.G228fs	Heterozygous	No
GT48	*BRCA2*	exonic	frameshift deletion	NM_000059	11	c.6402_6406del	p.N2134fs	Heterozygous	No
GT50	*ATM*	exonic	frameshift deletion	NM_000051	52	c.7702_7703del	p.R2568fs	Heterozygous	No

GT55	*FANCI*	exonic	frameshift deletion	NM_018193	33	c.3443_3444del	p.L1148fs	Heterozygous	No
GT64	*BRCA1*	exonic	frameshift deletion	NM_007297	9	c.3858_3861delTGAG	p.Ser1286Argfs	Heterozygous	No
GT7	*BRCA2*	exonic	stopgain	NM_000059	13	c.C6952T	p.R2318X	Heterozygous	No
GT80	*BRCA1*	exonic-intronic	larger deletion	NM_007297	9-12	-	-	Heterozygous	No
GT83	*CHEK2*	exonic	stopgain	NM_145862	10	c.1151delT	p.L384X	Heterozygous	No
GT84	*PTEN*	splicing	splicing	NM_000314	6	c.493-1G>T	-	Heterozygous	No
GT90	*NBN*	exonic	frameshift deletion	NM_002485	6	c.591_598del	p.Y197fs	Heterozygous	Yes
RR61	*RAD51C*	exonic	stoploss	NM_002876	2	c.T406C	p.X136Q	Heterozygous	No
T32	*BRCA1*	exonic	frameshift insertion	NM_007298	1	c.69_70insAG	p.C24fs	Heterozygous	No
T6	*RECQL4*	splicing	splicing	NM_004260	18	c.2885+1G>T	-	Heterozygous	Yes
T75	BRCA1	exonic	nonsynonymous SNV	NM_007297	16	c.C4982A	p.A1661E	Heterozygous	No
T81	*FANCM*	exonic	stopgain	NM_001308133	21	c.C5713T	p.R1905X	Heterozygous	No
T82	*BRCA2*	exonic	stopgain	NM_000059	10	c.C818A	p.S273X	Heterozygous	No
T85	*POLH*	exonic	frameshift substitution	NM_001291969	4	c.301_301delinsTT	p.L101fs	Heterozygous	Yes
T86	*PDE11A*	exonic	frameshift deletion	NM_016953	1	c.171delT	p.G57fs	Heterozygous	No
T99	*FANCF*	exonic	stopgain	NM_022725	1	c.C1087T	p.Q363X	Heterozygous	No

*Not described in ClinVar.

Table S2. Variants with unknown clinical significance detected in 327 patients

ID	Gene	Region	Type of change	Transcript	Exon	cDNA change	Protein change	Zygocity
**LN_1**	CHEK2	exonic	nonsynonymous SNV	NM_001349956	5	c.T506C	p.L169P	Heterozygous
**LN_11**	AIP	exonic	nonsynonymous SNV	NM_001302960	2	c.G145A	p.V49M	Heterozygous
**LN_35**	MSH2	exonic	nonsynonymous SNV	NM_000251	12	c.G1963A	p.V655I	Heterozygous
**LN_43**	AIP	exonic	nonsynonymous SNV	NM_001302960	2	c.G145A	p.V49M	Homozygous
**LN_50**	ATM	exonic	nonsynonymous SNV	NM_000051	4	c.A241G	p.N81D	Heterozygous
**LN_62**	CHEK2	exonic	nonsynonymous SNV	NM_001349956	9	c.G852T	p.E284D	Heterozygous
**LN_9**	EPCAM	exonic	nonsynonymous SNV	NM_002354	5	c.G518A	p.R173H	Heterozygous
**LN_EX1**	NF1	exonic	nonsynonymous SNV	NM_000267	30	c.C4009T	p.R1337W	Heterozygous
**LN_GT17**	BRIP1	exonic	nonsynonymous SNV	NM_032043	5	c.T415G	p.S139A	Heterozygous
**LN_GT207**	MSH2	exonic	nonsynonymous SNV	NM_000251	3	c.T581C	p.I194T	Heterozygous
**LN_GT216**	MUTYH	exonic	nonsynonymous SNV	NM_001350650	13	c.G976A	p.V326I	Heterozygous
**LN_GT221**	BRIP1	exonic	nonsynonymous SNV	NM_032043	7	c.C689T	p.S230L	Heterozygous
**LN_GT230**	BRCA2	exonic	nonsynonymous SNV	NM_000059	12	c.T6877C	p.F2293L	Heterozygous
**LN_GT235**	CHEK2	exonic	nonsynonymous SNV	NM_001349956	5	c.T506C	p.L169P	Heterozygous
**LN_GT24**	BRCA2	exonic	nonframeshift deletion	NM_000059	8	c.640_642del	p.214_214del	Heterozygous
**LN_GT250**	CDC73	exonic	nonsynonymous SNV	NM_024529	14	c.T1304C	p.M435T	Heterozygous
**LN_GT257**	MSH2	exonic	nonsynonymous SNV	NM_000251	12	c.G1963A	p.V655I	Heterozygous
**LN_GT27**	PTCH1	exonic	nonsynonymous SNV	NM_000264	23	c.G4027A	p.G1343R	Heterozygous
**LN_GT46**	MLH1	exonic	nonsynonymous SNV	NM_001167619	11	c.A413G	p.Y138C	Heterozygous
**LN_GT51**	CHEK2	exonic	nonsynonymous SNV	NM_001349956	7	c.A705C	p.E235D	Heterozygous
**LN_GT71**	MLH1	exonic	nonsynonymous SNV	NM_001258271	17	c.C1966T	p.R656C	Heterozygous
**LN_GT82**	RAD50	exonic	nonsynonymous SNV	NM_005732	13	c.C2173T	p.R725W	Heterozygous
**LN_GT82**	PALLD	exonic	nonsynonymous SNV	NM_001166108	3	c.C1040T	p.T347M	Heterozygous
**LN_GT85**	ATM	exonic	nonsynonymous SNV	NM_000051	27	c.C4060A	p.P1354T	Heterozygous
**LN_GT93**	BLM	exonic	nonsynonymous SNV	NM_000057	18	c.G3427A	p.E1143K	Heterozygous
**LN_GT93**	ATR	exonic	nonsynonymous SNV	NM_001184	41	c.T6961C	p.F2321L	Heterozygous
**LN_GT95**	PDGFRA	exonic	nonsynonymous SNV	NM_001347829	23	c.C3155T	p.T1052M	Heterozygous
**LN_GT95**	BLM	exonic	nonsynonymous SNV	NM_000057	3	c.A274G	p.N92D	Homozygous
**LN_GT95**	TMC6	exonic	nonsynonymous SNV	NM_001127198	20	c.G2368A	p.E790K	Heterozygous
**LN_GT97**	MLH1	exonic	nonsynonymous SNV	NM_001167619	12	c.G791A	p.S264N	Heterozygous

**LN_T16**	CHEK2	exonic	nonsynonymous SNV	NM_001349956	5	c.T506C	p.L169P	Heterozygous
**LN_T28**	FANCB	exonic	nonsynonymous SNV	NM_152633	3	c.T989C	p.I330T	Heterozygous
**LN_T30**	KDR	exonic	nonsynonymous SNV	NM_002253	26	c.C3439T	p.P1147S	Heterozygous
**LN_T35**	CHEK2	exonic	nonsynonymous SNV	NM_001349956	10	c.C1015T	p.R339C	Heterozygous
**LN_T38**	BLM	exonic	nonsynonymous SNV	NM_001287247	19	c.G3556A	p.E1186K	Homozygous
**LN_T46**	MUTYH	splicing	splicing	NM_001128425.1	14	c.1476+2G>A	-	Homozygous
**LN_T49**	PALLD	exonic	nonsynonymous SNV	NM_001166108	2	c.A731G	p.Q244R	Heterozygous
**LN_T69**	AXIN2	exonic	nonsynonymous SNV	NM_004655	2	c.C733T	p.P245S	Heterozygous

© 2018 by the authors. Licensee MDPI, Basel, Switzerland. This article is an open access article distributed under the terms and conditions of the Creative Commons Attribution (CC BY) license (http://creativecommons.org/licenses/by/4.0/).
